# The alteration of apoptosis-related genes in female pelvic supportive tissues with regard to menopausal status

**DOI:** 10.1007/s11033-023-09022-y

**Published:** 2023-12-12

**Authors:** Bahadır Saatli, Serap Kurt, Erkan Çağlıyan, Sefa Kızıldağ

**Affiliations:** 1 Department of Obstetrics and Gynecology, Dokuz Eylül University School of Medicine, Izmir, Turkey; 2 Department of Medical Biology and Genetics, Dokuz Eylül University School of Medicine, Izmir, Turkey

**Keywords:** Apoptosis, BCL-2 gene family, Menopause, Pelvic organ prolapse, Pre-menopause

## Abstract

**Purpose:**

We aimed to compare the expression levels of anti-apoptotic and proapoptotic genes in the parametrium, sacrouterine and round ligaments with respect to menopausal status in women presenting without any indication of pelvic organ prolapse (POP). We hypothesized that apoptosis related gene expressions in female pelvic tissues may be altered during menopause.

**Methods:**

The study groups consisted of pre-menopausal (n = 10) and menopausal (n = 10) females who did not have POP symptoms. Three different types of tissue samples (Parametrium, Round Ligament and Sacrouterine Ligament) were obtained and RNA was isolated from these tissues. After purifying and quantifying RNA samples, qPCR was used to determine the expression levels of anti-apoptotic and pro-apoptotic genes.

**Results:**

BCL-2 gene expression levels were significantly lower in all the tissues of menopausal patients compared to those of premenopausal patients. In comparison to premenopausal patients, the sacrouterine ligament tissue BAD expression level was significantly high (*p* = 0.035), and the BCL-2/BAD ratio was significantly lower in menopausal patients (*p* = 0.006).

**Conclusion:**

Apoptosis-related protein levels change during menopause; pro-apoptotic gene expressions decrease and anti-apoptotic gene expressions increase. The significant alteration of BCL-2 and BAD expression in sacrouterine ligament with respect to menopausal status was observed and this suggested that when compared to other pelvic tissues, the sacrouterine ligament, which plays a crucial role for genital organs in restoring normal pelvic anatomy and providing support, could be affected more by menopause.

## Introduction

The pelvic organs are supported by connective tissues attaching the uterus and vagina to the pelvic side wall and the levator ani muscles. Cardinal and uterosacral ligaments connect the cervix and upper part of the vagina to the pelvic walls. Commonly called the parametrium, the cardinal ligament on the lateral side of the uterus consists of vascular, neural, lymphatic and connective tissues. However, the uterosacral ligament supports the uterus posteriorly [1, 2]. The round ligament of the uterus consists of fibrous cords similar to the ones in the uterus body [3]. Cardinal and uterosacral ligaments comprise most of the supportive forces derived from the connective tissue of the female pelvic region [4]. Pelvic organ prolapse (POP) is a descent of the pelvic organs resulting in protrusions of the uterus, the vagina, or both. In one combination or another, it can affect the anterior and posterior walls of the vagina or uterus or apex of the vagina [5, 6]. It is more common in older women and those who have had multiple births [7]. Suggested risk factors for POP are multifactorial and include genetic predisposition, parity (particularly vaginal birth), menopause, estrogen deficiency, advancing age, prior pelvic surgery, connective tissue disorders, and factors associated with elevated intra-abdominal pressure. The cause of this disorder varies from patient to patient [8, 9]. Research has examined the disorders of the vaginal wall smooth muscles and connective tissues of women with POP. Accordingly, various studies have indicated that proteolytic activity and the apoptotic index in the extracellular matrix and apoptosis in the anterior vaginal wall were increased [10, 11].

Apoptosis is one of the mechanisms of cell death. The mitochondrial cell-death pathway involving the Bcl-2 family proteins Bcl-2, Bcl-XL, Bax, and Bad is one of the major pathways responsible for apoptosis [12–14]. Cells doubly deficient for the pair of pro-apoptotic molecules Bax and Bad proved resistant to all tested intrinsic death pathways [15]. In this pathway, the anti-apoptotic proteins are Bcl-2 and Bcl-XL, whereas the pro-apoptotic proteins are the Bax and Bad proteins. The balance between anti-apoptotic and pro-apoptotic genes is therefore critical in determining the ultimate apoptotic rate; the greater the expression level of pro-apoptotic genes, the more likely the cell will die [16–18]. Recent studies showed that apoptosis in pelvic tissues can be induced by different mechanisms including advanced glycation end products (AGEs) and insulin-like growth factor 1(IGF-1) [19, 20].

In our previous study, we compared the expression of apoptosis related genes in postmenopausal patients with or without POP and determined that Bcl-2 family genes were overexpressed in patients with POP [21]. In this current study, we aimed to define the effect of menopausal status on the expression of Bcl-2 family genes in pelvic connective tissues in patients without POP.

## Methods

### Determination of cases and tissue collection

The study was designed as a cross-sectional study. Twenty consecutive patients without any complaint of POP, ten premenopausal and ten postmenopausal women presenting at the Obstetrics and Gynecology Clinic of Medicine School of Dokuz Eylül University, were selected for the study. Benign disorders including benign ovarian cysts, dermoid cysts, paratubal cysts, ovarian fibromas, fundal intramural and pedunculated uterine fibromas were the indications for hysterectomy in both groups. Regardless of whether they had POP or not, all patients underwent a physical examination according to the International Pelvic Organ Prolapsa Quantification (POP-Q) system which is a subjective assessment for POP provided by the International Continence Society (ICS) [22]. Patients were excluded if they had gynecological cancer, cervical intraepithelial lesions, endometrial hyperplasia, endometriosis, connective tissue disorders, chronic disabling disorders, alcoholism, a smoking habit, or who had used hormonal replacement therapy. Ethical approval from the local ethics committee (date 14.12.2022, number 2022/40–16) and written informed consent from all patients were obtained for the study. Total abdominal hysterectomies with bilateral salpingo oophorectomy were performed in the menopausal group. In the premenopausal group, a total abdominal hysterectomy with bilateral salpingo oophorectomy was performed, however in cases which the ovaries were preserved, bilateral salpenjectomy was performed in addition to a hysterectomy. The materials used in the study consisted of parametrium, sacrouterine ligament, and round ligament tissues from premenopausal and postmenopausal patients. Tissue sampling was performed using Metzenbaum scissors for round and sacrouterine ligaments, a 10 scalpel was used for parametrial samples. Tissue samples were stored at − 80 ºC in tubes containing RNA and were subsequently delivered to the Dokuz Eylül University Medical Biology and Genetics Department.

### Total RNA extraction and cDNA synthesis

Tissue samples were obtained in 1.5 ml of micro centrifuge tubes containing RNA later (Qiagen, Hamburg, Germany). To obtain RNA from tissue, following homogenization of the tissues in the 1.5 ml of micro centrifuge tubes, RNA isolation was performed from the tissue using the ‘GeneAid Total RNA Isolation Mini Kit-Tissue in accordance with the manufacturer's instructions. The concentration and purity of the isolated total RNAs were determined using a spectrophotometer (NanoDrop™1000, Thermo Scientific, Waltham, MA, USA). Total RNAs whose purity and quantity were determined were converted to cDNA using ‘BioRad iScript Reverse Transcription SuperMix for RT-qPCR’ according to the manufacturer's instructions.

### Quantitative real time polymerase chain reaction (qRT-PCR)

In our study, a quantitative real-time polymerase chain reaction (qRT-PCR) was performed with GeneMarkBio qPCR SYBR Green kit in Qiagen Rotor-Gene device to determine the expression levels of anti-apoptotic and pro-apoptotic genes. Reactions were performed in a volume of 25 µl. The primers were purchased by the company in lyophilized form after being synthesized and purified by HPLC (Genosys Biotechnology Inc). The expression profile of BCL-XL, BCL-2, and BAD target genes and housekeeping HPRT gene as internal control were used in the qRT-PCR performed in this study. The primer sets for the genes are provided in Table 1. The same thermal profile was optimized for all primers: a pre incubation for 5 min. at 95 °C, followed by 40 amplification cycles of denaturation at 95 °C for 20 s, primer annealing at 58 °C for 30 s, and primer extension at 72 °C for 10 s. Melting curves were derived after 40 cycles by a denaturation step at 95 °C for 10 s, followed by annealing at 65 °C for 15 s, and a temperature rise to 95 °C with a heating rate of 0.1 °C/second and continuous fluorescence measurement. The final cooling was performed at 40 °C for 30 s. Melting curve analyses of each sample were accomplished using the LightCycler Software version 4.0.0.23 (Roche Diagnostics). All experiments were administered in triplicate. For relative quantification, the 2^ΔΔCt method was implemented [14].

**Table 1:** Primer sequences of related genes

HPRT	Forward	5′-GTGGAGATGATCTCTCAACT-3′
	Reverse	5′-ACATGATTCAAATCCCTGAAG-3′
BCL-XL	Forward	5′-GCTGGTGGTTGACT-3′
	Reverse	5′-GGATGGGTTGCCATTGA-3′
BCL-2	Forward	5′-TCAGGCTGCTTGGGATAAAGATG-3′
	Reverse	5′-GGCTTCTGGAGGACATTTGGA-3′
BAD	Forward	5′-GAGGTCCTGAGCCGACAG-3′
	Reverse	5′-CTTCCTCTCCCACCGTAGC-3′

### Statistical analysis

The results are expressed as median or mean ± standard deviation (SD). Comparisons between groups and within groups were performed using the Mann–Whitney U test or Two Way ANOVA. Differences were considered significant at *p* < 0.05. All statistical calculations were performed using ‘IBM SPSS Statistics V25’.

## Results

Biopsies were obtained from pre-menopausal (n = 10) and menopausal (n = 10) patients who underwent a hysterectomy and suffered no complications. There were no significant differences in body mass index (BMI) and parity between groups, though the median age was higher in the menopausal group. The clinical characteristics are provided in Table 2.

**Table 2:** The clinical characteristics of pre-menopausal and menopausal women without POP

	Pre-Menopausal (median) (n = 10)	Menopausal (median) (n = 10)	*p*-value
Age	43	52	0.002
Height (cm)	164.22	159.8	0.229
Weight (kg)	74.33	70.4	0.789
BMI	27.4	27.8	0.947
Parity	2.3	2.6	0.887

*BMI body mass index*

The expression levels of pro-apoptotic and anti-apoptotic genes were studied in pre-menopausal and menopausal women who had no symptoms of POP. When compared to menopausal women, anti-apoptotic BCL-2 gene expression was significantly higher in pre-menopausal women in all of the tissue samples examined and differences were statistically significant for all three tissues (parametrium *p* = 0.006, round ligament *p* = 0.003 and sacrouterine ligament *p* = 0.004). On the other hand, pro-apoptotic BAD gene expression levels for all tissues samples were higher in menopausal women when compared with premenopausal women. However, upregulation of BAD expression was only statistically significant for sacrouterine tissue (*p* = 0.035). The expression variations of anti-apoptotic and pro-apoptotic genes in different tissues are indicated in Table 3.

**Table 3:** Fold change in expression levels of anti-apoptotic (BCL-2 and BCL-XL) and pro-apoptotic (BAD) Bcl-2 family members in pre-menopausal and menopausal women without POP

Gene	Pre-Menopausal	Menopausal	*p*-value
Parametrium			
BAD	1.084 ± 0.100	9.855 ± 1.000	0.925
BCL2	114.115 ± 5.026	30.534 ± 1.500	0.006*
BCLXL	0.95 ± 0.150	1.061 ± 0.035	0.3
Round Ligament			
BAD	1.504 ± 1.500	1.729 ± 0.517	0.701
BCL2	127.82 ± 3.037	88.428 ± 3.111	0.003*
BCLXL	12.239 ± 1.560	7.88 ± 1.516	0.133
Sacrouterine			
BAD	4.996 ± 0.576	52.833 ± 1.512	0.035*
BCL2	114.854 ± 2.846	27.462 ± 1.035	0.004*
BCLXL	30.121 ± 1.542	47.645 ± 2.501	0.433

*Statistically significant

Gene expression profiles were also examined for the ratio of anti-apoptotic genes to pro-apoptotic genes. Accordingly, as indicated in Table 4, in comparison to menopausal women, the ratios of anti-apoptotic genes to pro-apoptotic genes in all tissues were determined to be higher in pre-menopausal women.

**Table 4:** Ratios of anti-apoptotic/pro-apoptotic gene fold change in expression levels in pre-menopausal and menopausal women without POP

Gene	Pre-Menopausal	Menopausal	*p*-value
Parametrium			
BCL2/BAD	427.869 ± 7.018	51.757 ± 1.091	0.096
BCLXL/BAD	13.066 ± 1.535	0.408 ± 0.377	0.947
Round Ligament			
BCL2/BAD	117.778 ± 4.130	116.192 ± 3.036	0.558
BCLXL/BAD	37.316 ± 1.572	9.019 ± 1.000	0.661
Sacrouterine			
BCL2/BAD	30.645 ± 1.021	0.924 ± 0.273	0.006*
BCLXL/BAD	4.055 ± 1.001	1.011 ± 0.500	0.739

*Statistically significant

**Fig. 1:**
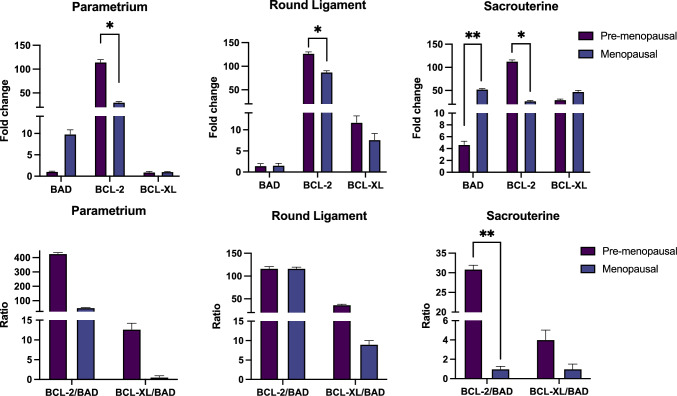
Fold changes in the expression of apoptosis-related genes in pre-menopausal and menopausal parametrium, round ligament and sacrouterine tissues

However, in pre-menopausal women, the BCL-2/ BAD ratio was significantly higher only in the sacrouterine tissue (*p* = 0.006). Fold changes in the expression of apoptosis-related genes in premenopausal and menopausal parametrium, round ligament and sacrouterine ligament tissues are provided in the Fig. 1.

## Discussion

In our study, the differences in the expression levels of anti-apoptotic (BCL-2, BCL-XL) and pro-apoptotic (BAD) genes were investigated in menopausal and premenopausal women without POP. In the literature, there are very few studies investigating the relationship of these target genes with regard to menopausal status.

The upregulation of anti-apoptotic BCL-2 gene expression in premenopausal patients was observed for all three tissue samples. In comparison to menopausal patients, these differences were statistically significant (parametrium *p* = 0.006, round ligament *p* = 0.003 and sacrouterine ligament *p* = 0.004). In a similar manner with regard to menopausal status, pro-apoptotic BAD gene expression levels for all tissues samples were higher in menopausal women when compared with premenopausal women. However, the upregulation of BAD expression, was statistically significant only for sacrouterine tissue (*p* = 0.035). BCL-2 levels were detected to be significantly higher in leiomyoma tissue in comparison to the normal myometrium and this may be correlated to the increased estrogen receptor levels in leiomyomas. A study was conducted by Xuxia Wu et al. which investigated the relation of BCL-2 levels between the leiomyoma and normal myometrium in relation to different hormonal status [23]. In this study, BCL-2 levels were found to be significantly higher in the proliferative phase of the menstrual cycle and premenopausal patients. This indicates that hormonal activity plays a crucial role in apoptosis balance with regard to circulating estrogen levels. In a similar study, BCL-2 expression levels were found to increase in the endometrial tissue of the proliferative phase in comparison with the endometrial tissue of the secretory phase in addition to the menopausal endometrial tissue as well. However, in the same study there was no significant differences in terms of myometrial tissues in different hormonal status in terms of BCL-2 levels [24]. It is a well-known fact that menopause has a negative effect on the uterus by mediating atrophy [25]. Shengtao Zhou et al. reported extensive decreased uterine tissue BCL-2 expressions in women with menopause [26]. Based on these findings and the results we observed, there is a correlation between estrogen levels and BCL-2 expression in pelvic tissue. In the absence of estrogen in the menopausal patients in our study, the anti-apoptotic action of BCL-2 is decreased. However, in premenopausal patients the expression of anti-apoptotic BCL-2 still continues. Consequently, it is possible to conclude that decreased estrogen levels are associated with the decreased anti-apoptotic action of BCL-2. On the other hand, the pro-apoptotic BAD expressions were increased by menopausal status in all the pelvic tissue samples we collected and indicated a statistically significant difference in the sacrouterine ligament.

Increased apoptotic activity in pelvic tissues may affect the development of pelvic floor disorders. Two of the well-known risk factors for POP are age and menopausal status [27]. Wen et al. conducted a study aimed to determine the expression change of the BCL-2 gene family in the vaginal tissues of women with pelvic organ prolapse. With this objective, anti-apoptotic and pro-apoptotic gene expressions were investigated by qRT-PCR and western blot analysis in vaginal tissue samples obtained from control and patient groups. The results indicated an increase in BAD and BAX expressions and a decrease in BCL-2/BAX and BCL-2/BAD ratios in cases with POP [28]. Similarly, in our study, anti-apoptotic BCL-2 and BCL-2/BAD ratios decreased in menopausal women compared to premenopausal women, and the expressions of BAD increased in all three tissue samples obtained from the menopausal patients. In addition, in our study, apoptosis markers were studied not only in one tissue sample but in samples from three tissues (the round ligament, sacrouterine ligament and the parametrium). Another study conducted to assess the apoptosis in POP patients revealed that pro-apoptotic Bax and Bad expression levels were significantly higher in sacrouterine ligaments of the POP patients compared to patients without POP [29]. In our study, BAD expression levels were found to be increased significantly only in the sacrouterine ligament of menopausal patients.

A recent study investigated the effect of estradiol on uterosacral ligament fibroblasts [30]. Estradiol treatment increased BCL-2 expressions in sacrouterine fibroblasts, generating a protective effect against apoptosis related cell death. We determined that estrogen absence (menopausal patients) decreased BCL-2 expressions in pelvic tissues. Additionally, pro-apoptotic BAD expression in the sacrouterine tissue increased significantly in menopausal patients. These findings enable us to state that menopause and the absence of estrogen decreased BCL-2 expression in pelvic tissues of women without POP. In contrast, pro-apoptotic BAD expression in the sacrouterine ligament increased. We surmise that the alteration of apoptosis related genes in the sacrouterine ligament plays a key role in the development of pelvic organ prolapse.

In our previous study, we investigated the relation of apoptotic markers between patients with and without POP, and observed that both pro-apoptotic and anti-apoptotic markers were significantly higher in patients with POP. This result suggested that increased cell turnover in POP may yield secondary increased anti-apoptotic markers (BCL-2, BCL-XL) in contrast to increased pro-apoptotic markers (BAX, BAD). In our present study, BCL-2 levels were significantly lower in all POP-related tissues including parametrium, round and sacrouterine ligament of menopausal patients in contrast with our previous study. Additionally, the detected sacrouterine ligament BAD level was higher and the BCL-2/BAD ratio was lower than the menopausal patients in the present study. These results are also compatible with our previous study [21].

The limitations of our study are the small sample size and the study design which did not allow us to analyze all factors effected by menopausal status. One strength of our study is that we collected only patients without POP so that we were able to analyze the effect of menopausal status (estrogen absence) on apoptosis related genes in different pelvic tissues. Another positive aspect is that we collected data only from patients without POP so that we were able to analyze the effect of menopausal status (estrogen absence) on apoptosis related genes in different pelvic tissues. Tissue samples (parametrium, round and sacrouterine ligament) were evaluated in terms of apoptotic factors.

In summary, anti-apoptotic and pro-apoptotic gene expression levels in different pelvic tissues were significantly altered in menopausal patients when compared with premenopausal patients without POP. Furthermore, anti-apoptotic BCL-2 expression levels decreased in menopausal patients in all the collected pelvic tissue samples suggesting that ageing and estrogen absence play a crucial role in pelvic tissue remodeling induced by menopausal status. Additionally, in comparison to the parametrium and round ligament, the sacrouterine ligament which plays a crucial role in restoring normal pelvic anatomy and providing support for genital organs, seems to be more affected by menopause. Further studies with larger sample sizes and including more determining factors are needed to present the characteristics of physiological pathways in female pelvic tissues by aging as a normal lifespan process. Subsequently, we can understand the mechanism of POP and develop preventive and therapeutic treatment option before POP occurs.

## Data Availability

The datasets used and/or analyzed during the current study are available from the corresponding author on reasonable request.
